# Functional neuroimaging of the interference between working memory and the control of periodic ankle movement timing

**DOI:** 10.1016/j.neuropsychologia.2013.07.009

**Published:** 2013-09

**Authors:** Leif Johannsen, Karen Z.H. Li, Magdalena Chechlacz, Attia Bibi, Zoe Kourtzi, Alan M. Wing

**Affiliations:** a Department of Sport and Health Sciences, Technische Universität München, Munich, Germany; b School of Psychology, University of Birmingham, Birmingham, United Kingdom; c Department of Psychology and Centre for Research in Human Development, Concordia University, Montréal, Canada; d Department of Experimental Psychology, University of Oxford, Oxford, United Kingdom

**Keywords:** fMRI, Dual-tasking, Ankle dorsi-plantarflexion, Hastening

## Abstract

**Background:**

Limited information processing capacity in the brain necessitates task prioritisation and subsequent adaptive behavioural strategies for the dual-task coordination of locomotion with severe concurrent cognitive loading. Commonly observed strategies include prioritisation of gait at the cost of reduced performance in the cognitive task. Alternatively alterations of gait parameters such as gait velocity have been reported presumably to free processing capacity for the benefit of performance in the cognitive task. The aim of this study was to describe the neuroanatomical correlates of adaptive behavioural strategies in cognitive-motor dual-tasking when the competition for information processing capacity is severe and may exceed individuals’ capacity limitations.

**Methods:**

During an fMRI experiment, 12 young adults performed slow continuous, auditorily paced bilateral anti-phase ankle dorsi-plantarflexion movements as an element of normal gait at .5 Hz in single and dual task modes. The secondary task involved a visual, alphabetic *N*-back task with presentation rate jittered around .7 Hz. The *N*-back task, which randomly occurred in 0-back or 2-back form, was modified into a silent counting task to avoid confounding motor responses at the cost of slightly increasing the task′s general coordinative complexity. Participants’ ankle movements were recorded using an optoelectronic motion capture system to derive kinematic parameters representing the stability of the movement timing and synchronization. Participants were instructed to perform both tasks as accurately as possible.

**Results:**

Increased processing complexity in the dual-task 2-back condition led to significant changes in movement parameters such as the average inter-response interval, the coefficient of variation of absolute asynchrony and the standard deviation of peak angular velocity. A regions-of-interest analysis indicated correlations between these parameters and local activations within the left inferior frontal gyrus (IFG) such that lower IFG activations coincided with performance decrements.

**Conclusions:**

Dual-task interference effects show that the production of periodically timed ankle movements, taken as modelling elements of the normal gait cycle, draws on higher-level cognitive resources involved in working memory. The interference effect predominantly concerns the timing accuracy of the ankle movements. Reduced activations within regions of the left IFG, and in some respect also within the superior parietal lobule, were identified as one factor affecting the timing of periodic ankle movements resulting in involuntary ‘hastening’ during severe dual-task working memory load. This ‘hastening’ phenomenon may be an expression of re-automated locomotor control when higher-order cognitive processing capacity can no longer be allocated to the movements due to the demands of the cognitive task. The results of our study also propose the left IFG as a target region to improve performance during dual-task walking by techniques for non-invasive brain stimulation.

## Highlights

• Neural correlates of involuntary ‘hastening’ of movements during cognitive-motor dual-tasking.
• Role of left inferior frontal gyrus and left superior parietal lobe in the temporal regulation of dual-task bilateral movements.
• Dissociation between left-hemisphere parietal involvement in external timing of bilateral movements and right hemisphere parietal involvement in interlimb coordination.

## 1 Introduction

Holding a conversation while walking is a form of multitasking which often occurs in normal daily life. In young and healthy individuals, talking and walking seem to involve distinct processes with minimum functional overlap and thus may appear especially effortless and undemanding in terms of conscious cognitive control. In contrast, control of gait parameters has been demonstrated to be associated with higher-order cognitive processes in older adults, supposedly to compensate for reduced automaticity in locomotion (Beauchet et al., 2011, Hausdorff et al., 2005, Springer et al., 2006). The increased fall risk with potentially dire consequences, expressed in terms of less precise movements and more variable gait (Hsu, Nagamatsu, Davis, & Liu-Ambrose, 2012), for example increased stride-to-stride time variability in single-task and dual-task situations (Beauchet et al., 2010), may stand in a direct relation to degraded automaticity of lower-order locomotor control centres and increased competitive demands for higher-order processing capacity in older adults (Lovdén, Schaefer, Pohlmeyer, & Lindenberger, 2008).

Adaptive behavioural strategies as a consequence of spontaneous task prioritisation when coping with inter-domain competition, benefitting performance in either the motor or the cognitive task, have been frequently described. For example, gait velocity, which seems unrelated to stride-to-stride time variability, occurs often as an important parameter adjusted by older adults in dual-task situations (Dubost et al., 2008). On the other hand, prioritisation of locomotion according to a ‘posture-first’ principle at the cost of exacerbated performance reductions in the cognitive task have been reported as well (Li, Lindenberger, Freund, & Baltes, 2001). Although these adaptive strategies have been observed predominantly in older adults, there is no reason to believe that they will not occur in younger adults too, provided significant competition for higher-order processing capacity between the motor and cognitive domains.

Performance costs generally observed during dual-tasking have been taken to indicate some kind of process interference, for example either based on limitations of a graded attentional resource, processing ‘bottlenecks’ due to sharing of a central capacity or structural interference resulting from simultaneous involvement of specific neural circuitry in both tasks (Pashler, 1994). The source of cognitive-motor competition in dual-task walking is not entirely clear, although many behavioural studies signify competition for processes represented in the prefrontal cortex. For example, an association between gait variability and measures of executive control is suggested by studies correlating gait parameters with neuropsychological test scores in older adults (Beauchet et al., 2011, Hausdorff et al., 2005, Springer et al., 2006). A recent behavioural study by Yogev-Seligmann, Giladi, Gruendlinger, & Hausdorff, 2013 suggested that the effects of cognitive load on walking result from a susceptibility of bilateral coordination to dual-tasking and not from the demands of stabilising body balance.

In terms of brain function, attempts have been made to describe the neural correlates of actual walking under dual-task conditions by means of functional near-infrared spectroscopy (fNIRS). Holtzer et al. (2011) used fNIRS to demonstrate that overground walking while talking reduces gait velocity and increases prefontal cortical activations in both young and older adults. Nevertheless, their study leaves a number of questions unanswered as no parameters other than gait velocity were reported, thus precluding distinctions between postural and temporal contributions to the pattern of brain activity. In addition, the spatial resolution was low and the sensor coverage of the brain was limited which makes differentiation of specific brain areas within the prefrontal cortex and elsewhere in the brain difficult.

To summarise, the demands of walking in terms of spatiotemporal gait adjustments, bilateral coordination and control of body balance are likely to require the involvement of executive control circuits represented in the prefrontal cortices. Walking while performing a concurrent cognitive task, which also demands executive control such as a working memory task, invokes competition for central processing capacity with results in the need for spontaneous task prioritisation and an adequate adaptive behavioural strategy. What remains unresolved in particular is the susceptibility to dual-task interference of cognitive processes involved in the temporal control of gait as well as the question whether a methodology with higher spatial resolution than fNIRS such as fMRI will be able to distinguish between the respective neural correlates.

### 1.1 The current study

The present study pursued three major aims. The first was to isolate the neural correlates of gait timing in young adults. The second was to identify in a region of interest analysis potential compensatory brain activations that contribute to the maintenance of gait timing under heavy dual-task loading. The third aim was to invoke process competition within prefrontal cortical regions that simulates age-related reductions in cognitive processing capacity for dual-tasking. In contrast to Holtzer et al. (2011), our study investigated activation across the entire brain, covering the cerebrum and the cerebellum, and registered movement parameters representing timing and coordination of movements. In addition, our study isolated the temporal as well as the coordinative aspects of dual-task walking from the postural aspects by relating brain activations directly to motor performance.

Our approach was to assess the supra-spinal correlates of walking without imposing the same biomechanical and postural demands by asking participants to perform bilateral ankle dorsi-plantarflexion movements. Dobkin, Firestine, West, Saremi, and Woods (2004) suggested that fMRI of ankle dorsiflexion might serve as a valid task for studying the supraspinal correlates of sensorimotor control of walking. We aimed to create a situation where the processing requirements for each task were considerable but simultaneous execution still possible. We expected that without the need to consider postural stability (‘posture-first’ principle) the CNS would search for an optimised strategy for the coordination of the two concurrent tasks. Therefore, both our motor and cognitive tasks were designed to involve continuous processing, which might cause intermittent load on a central processing capacity but which might also be compensated for by appropriate task scheduling (e.g., Broadbent, 1982). For the cognitive demands we selected an *N*-back paradigm with low (0-back) and high (2-back) coordinative complexity and we combined these two tasks with continuous bilateral ankle movements in the dual-task conditions.

Behaviourally we expected to find that dual-task load should negatively affect the timing accuracy and periodicity of bilateral ankle dorsi-plantarflexion movements. We expected to see these interference effects on motor behaviour specifically in the one condition imposing high cognitive load. Costs in cognitive performance might or might not occur depending on whether participants would spontaneously prioritise the cognitive domain.

With respect to the BOLD activity during dual-tasking, we expected to see a distribution of brain activations that would result from the combination of the two single-task conditions. For example, an alphabetical *N*-back task should result in a fronto-parietal network involving the left-hemisphere dorsolateral prefrontal cortex (Collette and Van der Linden, 2002, Collette et al., 2006), while the motor task should activate its respective bilateral primary sensorimotor cortex, bilateral supplementary motor areas and the bilateral cerebellum (Swinnen and Wenderoth, 2004, Walsh et al., 2008). Specific areas that are associated with these single-task networks might show increased activations as a function of increasing coordinative complexity in order to meet dual-task requirements (Smith & Jonides, 1997). Alternatively, additional specific dual-task-related activations might be found (Cohen et al., 1997). As for the novel combination of a cognitive with a motor task, we could not predict whether we would find a pattern of upregulation or see an emergence of supplementary areas.

Finally, given that cognitively controlled timing has been shown to rely on bilateral fronto-parietal networks normally representing working memory and attentional processes (Lewis & Miall, 2003), we expected the dual-task combination of the *N*-back and the movement tasks to induce structural interference within these regions. In order to establish a more direct link between neural correlates and motor performance, we subjected relevant brain regions of interest (ROIs) activated during dual-tasking to correlational analyses with the behavioural movement data. Therefore, in terms of a structural interference model, we expected neural structures associated with motor timing to correlate positively with accurate and consistent movement.

## 2 Methods

### 2.1 Participants

Twelve healthy young adult participants (mean age =26.1 yrs, SD=4.7, range 21.7–37.8; 8 female, 4 male) volunteered for the study. All reported themselves as right-handed for writing and correspondingly reported their right foot as the dominant for lower limb activities such as kicking a ball. An entry screening procedure and questionnaire ensured that participants were aware of all critical health and safety issues associated with fMRI. Individuals reporting intoxication or use of psychotropic substances were excluded from the study. The study design was approved by the University of Birmingham Ethics Committee and written informed consent was obtained from all participants in agreement with the ethical protocol at the Birmingham University Imaging Centre (BUIC).

### 2.2 Experimental block design

Participants took part in two experimental sessions typically scheduled on two separate days within the same week. On the first day participants practiced the full experimental protocol comprising a minimum of 4 experimental blocks (52 trials), including movement data acquisition, inside the mock scanner at BUIC. On this occasion only, they received written and verbal instructions, gained live experience in each of the experimental conditions and became familiar with the task colour scheme which was used to indicate each experimental condition. On the second day, participants entered the scanner suite for the main assessment session and performed at least 4 experimental blocks. In order to increase the trial numbers per participant, additional experimental blocks were acquired if participants indicated sufficient concentration and motivation to continue (2 participants completed 4 blocks, 9 participants completed 5 blocks, one participant, 6 blocks).

A partly randomized experimental design was implemented in which a single experimental block consisted of 13 trials, each trial of 42 s duration. Every 1st, 7th and 13th trial in a block provided data for a fixation-rest condition. The remaining 10 trials (trials 2–6 and 8–12) were split into two mini-blocks of 5 trials each. Each mini-block contained one trial from each of the five experimental conditions in randomized order. Before each trial began, an inter-trial interval of two seconds maximum duration ensured synchronization between brain volume image data acquisition and trial commencement. These intervals were also used to display in writing the upcoming experimental condition to the participants. All experimental trials, including the fixation-rest condition, consisted of the simultaneous administration of a sequence of letter stimuli and a sequence of regular auditory pulses. An entire block took slightly more than 10 min to complete.

### 2.3 Stimulus conditions and trial types

Two of the three single-task conditions utilised a modified alphabetic *N*-back paradigm in which a sequence of 29 individual letters was presented in RED at the centre of the screen in font size 96. Each letter was visible for a constant 1229 ms followed by a blank screen. To reduce entrainment to the cognitive task, the blank screen duration was randomly jittered within a range of 120–280 ms, averaging at 200 ms. The visual presentation rate approximated .7 Hz.

**Fig. 1:**
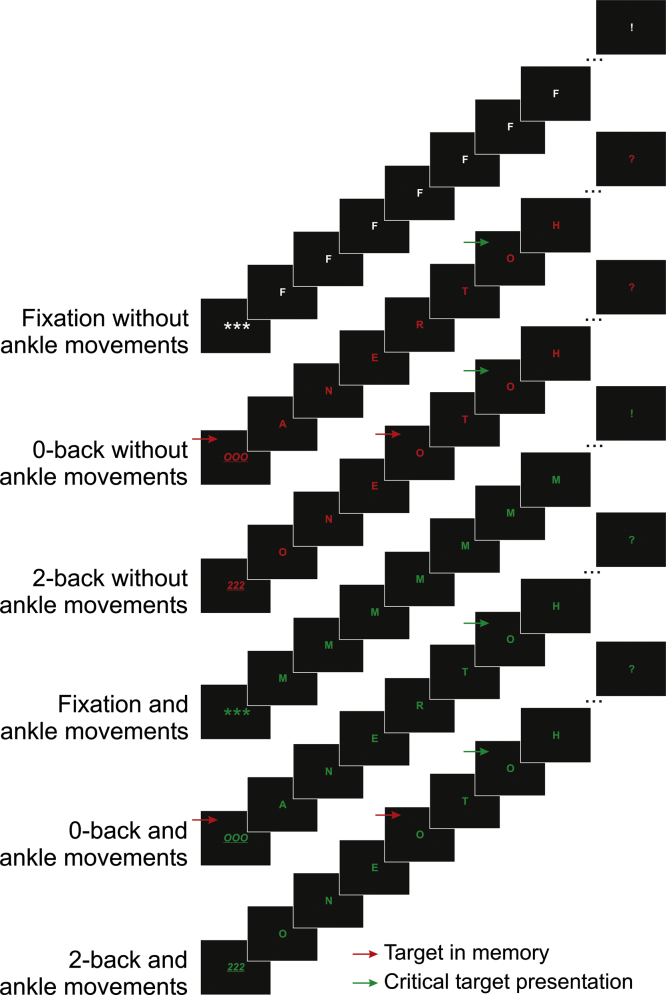
Overview of the 6 types of experimental trials. (For interpretation of the references to colour in this figure caption, the reader is referred to the web version of this article.)

The *N*-back tasks were performed with either low (0-back) or high (2-back) memory load. During the 0-back condition (no WM updating), which controlled for general dual-tasking effects, the very first visual presentation indicated a single random letter as the search target for the subsequent alphabetical sequence (see Fig. 1). To avoid any possible form of interference between the periodic movements and a discrete manual response, participants were instructed to silently count the number of target repetitions. At the end of the presentation period, participants reported the number of targets counted by button press on one of the two button boxes under each hand. The response had to be entered within three seconds, as indicated by a central question mark, or would be registered as a missing value. The response key and latency were recorded. Participants were asked to guess the correct number of targets if they were uncertain. For the 2-back trials (continuous updating), participants were cued (see Fig. 1) to count the number of occasions on which the currently presented letter was identical to the letter presented two items prior in the sequence. In both *N*-back conditions, participants were aware that the randomised target count ranged from 0 to 6 possible targets (chance rate=1/7=14%).

For the third single-task condition, during which bilateral ankle movements were performed exclusively, a random letter was repeated 29 times in green to equate the visual demands of the single- and dual-task conditions. Participants were instructed to perform slow periodic bilateral ankle dorsi-plantarflexion movements in opposite directions. They did not receive any specific instructions regarding ankle movement amplitude. Participants were instructed to synchronise the end points of full ankle movement cycles to a regular auditory pacer stimulus (6000 Hz beep of 15 ms duration) set to a 2 s inter-stimulus interval (.5 Hz). In behavioural pilot studies comparing 1 Hz movement periodicity against .5 Hz we found that cognitive–motor interference effects on cognitive and movement parameters were more pronounced at the slower .5 Hz movement rate. Closed-caption TV was used to check for any extraneous movements.

Participants' knees were raised by inserting a wedge cushion under the lower legs so that the distal segments were oriented 45° downwards. Another pillow was inserted under the lower leg just above the ankle to prevent the heel of a foot from touching either the wedge cushion or the scanner bed. This arrangement was chosen to allow for unconstrained movements of the ankles while reducing head motion artefacts.

In the two dual-task conditions, the 0-back and 2-back tasks were combined with the motor task. Participants were instructed to perform as best as they could in both tasks, thus implying equal task priority: in the *N*-back task in terms of the overall accuracy, and in the motor task in terms of the movements‘ smoothness and spatiotemporal regularity.

Finally, participants were required to remain passive while perceiving the auditory and visual stimuli in the fixation-rest condition. As in the single-task movement condition, one specific randomly drawn letter presented in white was repeated 29 times. Fig. 1 provides an overview of the 6 types of trials within an experimental block.

### 2.4 Apparatus

Ankle movement kinematics were recorded at 200 Hz throughout the whole experiment using an fMRI-compatible optoelectronic motion capture system consisting of 4 cameras (Qualisys Profreflex, Sweden). Four passive reflective markers were placed on each foot to mark the toe, the lateral ankle, the mid-point between the inner and outer ball of the foot, and the midpoint of the longitudinal arch. These four markers and their trajectories provided sufficient redundancy for reconstruction of ankle dorsi- and plantarflexion movements. A stimulus program was written in Matlab 7.5 (MathWorks, Natick, MA, USA) for the delivery of the visual and auditory stimuli using the Psychophysics Toolbox extensions (Brainard, 1997, Pelli, 1997). This program ran on a separate computer and synchronized the start of each presentation sequence in an experimental trial with the trigger pulse provided by the brain scanner at the beginning of the image data acquisition for each single brain volume. The program also provided analog pulses indicating the onset time of each visual presentation event and metronome pulses to a single multiplexed analog to digital converter (ADC) connected to the motion capture system. Consequently, the onset of the first metronome pulse was used for triggering the kinematic and analog (at 1200 Hz) data acquisition by the motion capture system.

### 2.5 Kinematic data processing and data reduction

**Fig. 2:**
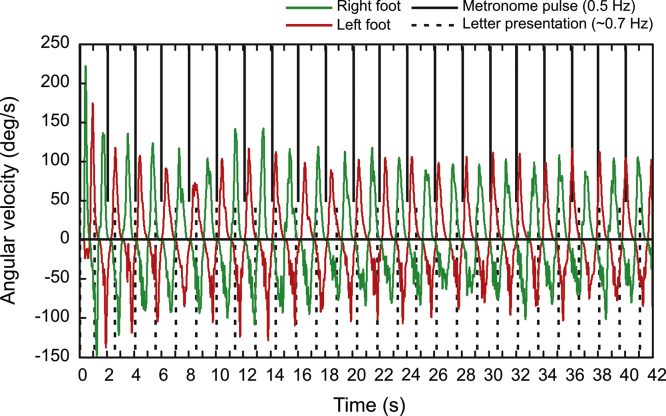
An illustrative trace of angular velocity during dorsi-plantarflexion of both ankles for an entire trial of a single individual. The onset of the auditory pacing stimulus is indicated by the top axis of the figure, while the onset of the *N*-back stimuli is plotted along the bottom axis.

All data processing was done in Matlab 7.5. Kinematic time series data were upsampled from 200 Hz to 1200 Hz to match the sample rate used for recording the visual presentation event and metronome pulses. The upsampled kinematic data were smoothed using a moving average with window width equivalent to 100 ms. Cartesian coordinates of the toe markers were transformed into Polar coordinates with the centre of rotation as the origin derived from the toe markers’ curved trajectories. A vertical line through the origin served as the 0° reference angle for the calculation of the movement angles. Movement angles were differentiated to yield angular velocity of foot movements. Peak angular velocity time points were detected for both feet and every movement cycle in an experimental trial that required ankle movements. For each of these trials, the maximum range of ankle dorsiplantar flexion in terms of the peak to peak amplitude was determined as well. Fig. 2 shows angular velocity for both ankles over an entire trial from a single individual.

In order to quantify participants' movement timing, relative and absolute asynchrony of velocity peaks was measured in relation to the closest onset of a metronome pulse. The elapsed time between successive angular velocity peaks was termed the ‘inter-response interval’ (IRI). The continuous relative phase between the angular velocity profiles of both feet was computed in order to estimate participants' interlimb coordination using an algorithm that utilised the Hilbert transform (Rosenblum & Kurths, 1998).

### 2.6 Statistical analysis of behavioural data

Proportion of correct responses quantified participants' performance in the *N*-back task under single and dual-task conditions, while their motor performance was summarised with the following movement parameters: Average (AV) and standard deviation (SD) of peak velocity, coefficient of variation (CV) of the absolute movement asynchrony, average and CV IRI, and average and SD continuous relative phase (CRP). For the motor performance outcome variables, a repeated-measures ANOVA was used with experimental task condition (single-task motor, dual-task motor and 0-back load, dual-task motor and 2-back load) as a within-subjects factor. Movement parameters were averaged across both feet within each participant, as preliminary analyses did not show any effects of body side. The proportion of correct cognitive responses was analyzed using a repeated-measures ANOVA with coordinative complexity (single-task vs dual-task coordination) and working memory load (0-back vs 2-back) as within-subject factors. All statistical analyses were performed in SPSS 16 (IBM Corporation, Somers, NY, USA) with significance levels set at *p*=.05 after Greenhouse-Geisser correction.

### 2.7 Brain volume image data acquisition

Participants were scanned on a 3T Philips Achieva MRI system. Images were acquired using an 8-channel phased array head coil with a SENSE factor of 2. For a high resolution anatomical scan, a sagittal T1-weighted sequence (sagittal orientation, echo time/slice repetition time, TE/TR=3.8/8.4 ms, voxel size 1×1×1 mm^3^) was used. For the functional scans, 299 BOLD contrast-weighted echo-planar image volumes (EPIs) were acquired for each experimental block. In-plane resolution was 2^⁎^2^⁎^3.75 mm with 35 ms TE and 2 s TR.

### 2.8 Image data processing and analysis

Imaging data were analyzed in SPM8 (www.fil.ion.ucl.ac.uk/spm; Wellcome Department of Imaging Neuroscience, London, UK) running in Matlab 7.5. For every participant, EPI volumes for each experimental block were spatially realigned to the first image volume in a block and the corresponding realigned mean image volume was created. The realigned image volumes were unwarped in order to remove signal variance that might have been caused by unwanted head movements. The mean image volumes were transformed to the MNI standard space and the resulting normalisation parameters were applied to the realigned-unwarped image volumes. Normalised images were smoothed with an isotropic Gaussian kernel of 8 mm FWHM. The final image volume resolution of the resampled images was 2 mm^3^.

A General Linear Model approach was followed for the 1st subject-level analysis during which every experimental condition was modelled by a convolution between a box-car function with the SPM hemodynamic response function. Low frequency signal drift with a period longer than 128 s was removed from the image data and a first-order autoregressive model was used to account for serial correlations in the image volume time series. Finally, contrast images were calculated between each of the 5 experimental task conditions and the fixation-rest condition.

### 2.9 Region of interest analysis

As the dual-task 2-back condition showed statistical effects on specific movement parameters compared to the single-task motor and the dual-task 0-back conditions, we identified those clusters in the dual-task 2-back 2nd level main effects with a cluster significance level below .05 after false discovery rate (FDR) correction for multiple comparisons as our selected Regions of Interest (ROI). On the 1st level contrast images, we used the MarsBar toolbox (Brett, Johnsrude, & Owen, 2002) to centre spheres of 3 mm radius at the respective MNI coordinates of each local peak and to average the mean beta sum across all voxels within a particular sphere for every participant. The anatomical structure at the coordinates of a respective local peak was identified using the Jülich probabilistic cytoarchitectonic maps (Anatomical map V18; Zilles et al., 2002). Finally, across all 12 participants, non-parametric Spearman’s Rho correlation coefficients and linear regression parameters were calculated between a sphere′s averaged beta sum and the average of each of the outcome movement parameters using SPSS 16. For each local peak, post-hoc comparisons between the three task conditions were calculated for the average activation within its respective volume of interest using a repeated-measures ANOVA with a Bonferroni-adjusted alpha-level of .001 (.05/(3^⁎^16)=.001042).

## 3 Results

### 3.1 Behavioural findings

**Fig. 3:**
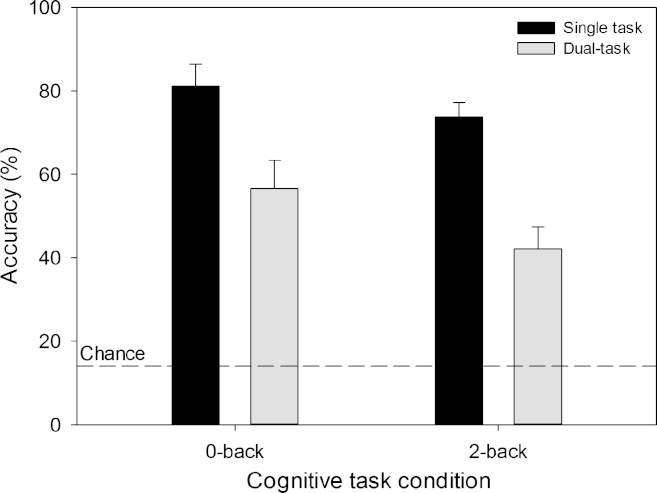
Accuracy of *N*-back task performance in the single-task and dual-task conditions. The error bars indicate the standard error of the mean.

Fig. 3 shows the accuracy for the two *N*-back tasks in both the single-task and dual-task conditions. Accuracy was negatively affected by increases in coordinative complexity [single- vs dual-task; *F*(1,11)=13.88, *p*=.003, partial *η*^2^=.56] and memory load [0-back vs 2-back; *F*(1,11)=6.23, *p*=.03, partial *η*^2^=.36]. No interaction between the two factors was found. Accuracy was highest in the 0-back single-task condition and lowest in the dual-task 2-back condition.

We first examined the maximum range of ankle movements and determined that they did not differ between task conditions or body side. The maximum range of ankle movements was 51.7° (SD 9.0). In terms of movement speed, peak angular velocity of the ankle movements was 131.2°/s (SD 23.5) and again no significant effect was found between task conditions. In contrast, the within-trial variability (SD) of peak velocity differed between the three task conditions [*F*(2,22)=4.07, *p*=.05, partial *η*^2^=.27]. Post-hoc comparisons traced this effect to a significant reduction in SD peak velocity during the dual-task 2-back condition (mean=23.9°/s, SD 8.0) relative to the single-task [mean=26.3°deg/s, SD 9.3; *F*(1,11)=5.97, *p*=.03, partial *η*^2^=.35].

We next examined movement timing and periodicity to assess the effects of cognitive load on the temporal aspects of the motor task most analogous to the temporal aspects of gait. The coefficient of variation (CV) of absolute asynchrony differed between the task conditions [*F*(2,22)=10.91, *p*=.001, partial *η*^2^=.50]. Post-hoc comparisons showed that participants exhibited greater variability in the dual-task 2-back condition (mean= 43.1%, SD 10.0) than in either the dual-task 0-back (mean= 34.7%, SD 10.1) or the single-task condition [mean=32.0%, SD 8.7; both *F*(1,11)>11.34, both *p*<.006, both partial *η*^2^>.51].

**Fig. 4:**
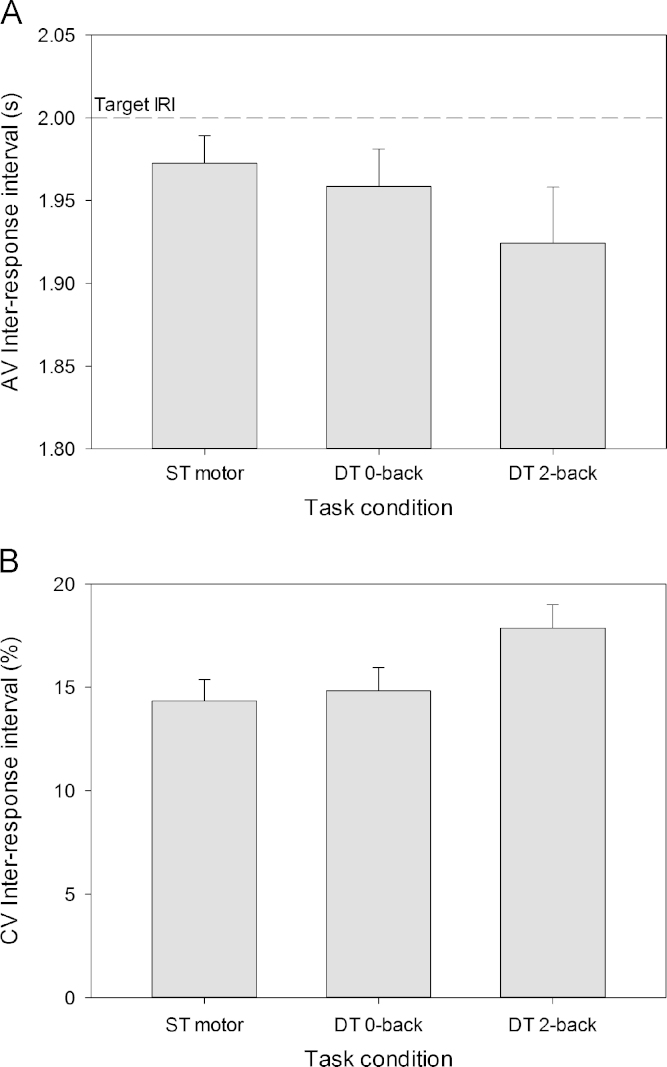
(A) AV movement inter-response interval and (B) CV IRI as a function of task condition. The error bars indicate the standard error of the mean. ST, single-task; DT, dual-task.

Fig. 4 shows the average and CV of the movement inter-response interval as a function of the task condition. The inter-response interval showed a marginal effect of task condition [*F*(2,22)=4.23, *p*=.06, partial *η*^2^=.28]. Post-hoc comparisons showed that the inter-response interval tended to be shorter in the dual-task 2-back condition compared to the single-task and dual-task 0-back conditions [both *F*(1,11)≥4.53, both *p*≤.06, both partial *η*^2^≥.29]. Likewise, CV IRI showed a main effect of task condition [*F*(2,22)=10.65, *p*=.002, partial *η*^2^=.49]. Post-hoc comparisons indicated that the dual-task 2-back condition resulted in more variable CV IRI compared to the other two tasks [both *F*(1,11) ≥11.30, both *p*≤.006, both partial *η*^2^≥.51].

A final aspect of motor performance that we examined was bilateral interlimb coordination, as indexed by average CRP between the two feet. This measure was affected by the task condition [*F*(2,22)=10.06, *p*=.001, partial *η*^2^=.48]. Post-hoc comparisons confirmed that all three conditions differed from each other [all *F*(1,11) ≥4.69, all *p*<.05, all partial *η*^2^≥.30), which indicates that a shift in the anti-phase relationship between the two feet occurred from a slight lead of the dominant foot in the single-task condition to a slight lead of the non-dominant foot with increasing dual-task demands. SD CRP, on the other hand, was not different between the task conditions. Cognitive accuracy correlated negatively with SD CRP in the dual-task 2-back condition (*r*=−.64, *p*=.02) but not in the dual-task 0-back condition. Thus, a reduction in the accuracy correlated with an increase in the variability of interlimb coordination under dual-task 2-back load.

### 3.2 Task-dependent brain activations

Table 1 lists local peaks of significant clusters activated in the 2nd level single-task motor and 2-back conditions. Activations in the single-task motor condition were distributed bilaterally across regions of the brain well known to be involved in foot movements such as the primary sensorimotor cortex and posterior supplementary motor area of the paracentral lobule, the anterior cerebellum, the superior temporal gyrus, the insula and the basal ganglia. Activations in the single-task 2-back condition were also spread bilaterally within the frontal and parietal lobes.

**Table 1:** Significant local peak activation in the single-task motor and 2-back conditions.

Single-task motor						Single-task 2-back					
Area	Side	MNI coordinates			Local peak	Area	Side	MNI coordinates			Local peak
		L–R (*X*)	A–P (*Y*)	I–S (*Z*)	*t*			L–R (*X*)	A–P (*Y*)	I–S (*Z*)	*t*
Cerebellum (lobules I–IV)	L	−12	−40	−24	8.83	Middle frontal gyrus	L	−24	−4	50	8.36
Cerebellar vermis		0	−44	−16	11.86	Middle frontal gyrus	R	30	2	56	5.15
Cerebellum (lobules I–IV)	R	16	−38	−26	11.06	Middle frontal gyrus	R	42	42	18	4.98
Paracentral lobe	L	0	−14	70	10.76	Middle frontal gyrus	R	34	34	22	4.34
Paracentral lobe	L	−4	−36	66	10.64	Inferior parietal lobe	L	−34	−44	40	8.60
Paracentral lobe	R	6	−34	68	10.64	Superior parietal lobe	L	−26	−62	44	8.59
Superior temporal gyrus	L	−46	−32	16	5.79	Superior parietal lobe	L	−26	−68	56	7.62
Superior temporal gyrus	L	−42	−34	8	4.95	Supramarginal gyrus	R	44	−36	42	6.55
Superior temporal gyrus	R	50	−36	16	6.38	Angular gyrus	R	32	−46	40	6.37
Superior temporal gyrus	R	60	−30	16	5.89	Angular gyrus	R	28	−62	42	5.69
Insula	R	42	0	0	5.41	Precentrial gyrus	L	−42	−2	54	7.57
Caudate nucleus	R	16	−16	20	6.06	Precentrial gyrus	L	−50	8	34	6.67
Caudate nucleus	R	16	−6	20	5.97	SMA	L	−4	8	54	7.03
Supramarginal gyrus	L	−60	−30	26	4.50	Insula	R	34	22	−4	4.85
Precentral gyrus	L	−58	6	26	4.94						
Temporal pole	L	−42	12	−20	4.54						

L, left hemisphere; R, right hemisphere; L–R, left-right; A–P, anterior–posterior; I–S, inferior–superior; SMA, supplementary motor area.

**Fig. 5:**
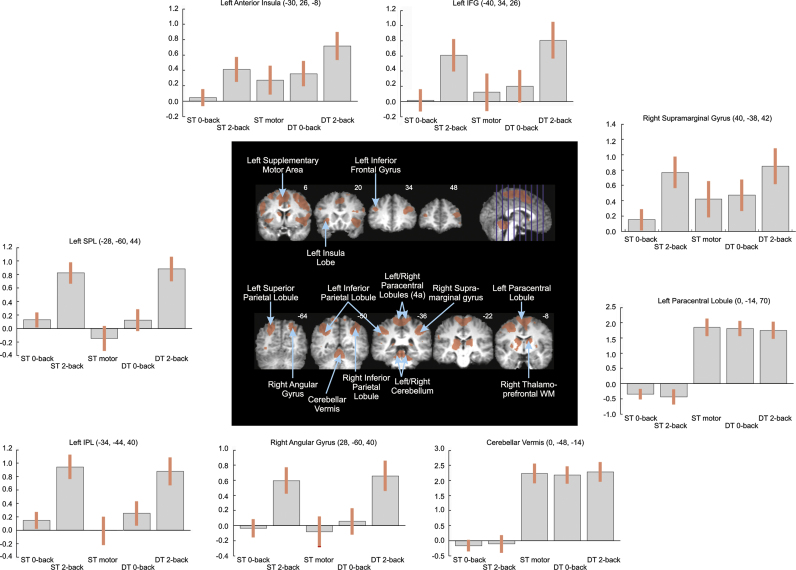
Distribution of 2nd level main effect activations for the dual-task 2-back condition. Local peaks of significant clusters are labelled. Surrounding panels represent contrast patterns for selected clusters. ST, single-task; DT, dual-task.

Fig. 5 shows the distribution of 2nd level main effect activations for the dual-task 2-back condition. Large parts of the distribution of brain activations found in this condition were very similar to the single-task motor and dual-task 0-back conditions which argue in favour of motor-related activity within those respective regions. Compared to the single-task 2-back condition, we found very similar bilateral symmetrical activations within the frontal and parietal lobes.

In order to detect any dual-task specific activations, we contrasted each of the two dual-task conditions with the sum of the corresponding two single-task conditions (Szameitat, Schubert, & Muller, 2011) but did not find any significant clusters. Lowering the cluster significance threshold to a less conservative level (uncorrected *p*<.05) also did not yield any significant local BOLD activations. Finally, we did not observe any significant BOLD deactivations in either of the two dual-task conditions relative to the respective sum of the single task conditions. This was true for both the more conservative FDR-corrected cluster level and the more lenient level of uncorrected *p*<.05.

Table 2 lists 16 local peaks of 6 significant activation clusters in the dual-task 2-back condition. Post-hoc comparisons indicated two major types of contrast pattern (Fig. 5). The first pattern was that ROIs within motor-related areas such as the primary motor cortex and the cerebellum did not show any differences across the single-task motor, dual-task 0-back, and dual-task 2-back conditions, while showing no activations in the 2 cognitive single-task conditions. The second notable pattern involved stronger local activations in the dual-task 2-back condition compared to the dual-task 0-back and single motor condition within the inferior frontal gyrus, the superior parietal lobule and the inferior parietal lobule in the left-hemisphere and within the parts of the angular gyrus in the right hemisphere.

**Table 2:** Significant local activation peaks in the dual-task 2-back condition.

Area	Side	MNI coordinates			Local peak	Task condition contrasts		
					*t*	DT2b vs STm	DT2b vs DT0b	DT0b vs STm
		L–R (*X*)	A–P (*Y*)	I–S (*Z*)		*F*(1,11)		
Anterior insula	L	−30	26	−8	6.46	21.97⁎	−	−
Anterior insula	L	−34	14	−4	6.10	−	−	−
Thalamus	R	16	−14	18	6.37	−	−	−
Inferior frontal gyrus	L	−40	34	26	5.49	25.25⁎	31.42⁎	−
Cerebellum (lobules I-IV)	L	−10	−42	−22	9.56	−	−	−
Cerebellar vermis		0	−48	−14	11.70	−	−	−
Cerebellum (lobules I−IV)	R	16	−38	−28	11.82	−	−	−
Paracentral lobe	L	0	−14	70	10.35	−	−	−
Paracentral lobe	L	−4	−38	66	10.26	−	−	−
Paracentral lobe	R	8	−34	70	10.34	−	−	−
Superior parietal lobe	L	−28	−60	44	8.10	45.76⁎	43.19⁎	−
Inferior parietal lobe	L	−50	−38	48	5.74	−	−	−
Inferior parietal lobe	L	−34	−44	40	7.03	43.61⁎	25.62⁎	−
Angular gyrus	R	28	−60	40	5.49	39.05⁎	52.21⁎	−
Inferior parietal lobe	R	34	−48	40	6.07	−	−	−
Supramarginal gyrus	R	40	−38	42	6.04	−	−	−

⁎ *p*<.001; DT2b, dual-task 2-back; DT0b, dual-task 0-back; STm, single-task motor; L, left hemisphere; R, right hemisphere; L–R, left–right; A–P, anterior–posterior; I–S, inferior–superior.

Table 3 lists the two local peaks that showed significant correlations between their respective local activations and at least one specific movement parameter in the dual-task 2-back condition. The left-hemisphere inferior frontal gyrus stands out as a ROI where local activations showed strong correlations with average IRI, CV of the absolute asynchrony and SD peak velocity in the dual-task 2-back condition, while no correlations between movement parameters and activations were present in the dual-task 0-back or single-task conditions. An apparent dependency between these three movement parameters is also evidenced by significant correlations among the movement parameters themselves exclusively in the dual-task 2-back condition [average IRI with SD peak vel: *r*=.64; average IRI with CV absolute asynchrony: *r*=−.76; CV absolute asynchrony with SD peak vel: *r*=−.77; all *p*<.05].

**Table 3:** Significant correlation between movement parameters and activations in two local ROIs showing increased activations in the dual-task 2-back condition.

Area	Side	MNI coordinates			Task condition	Coefficient of correlation (*R*)			
						AV IRI	SD peak vel	CV asynch	SD CRP
		L–R (*X*)	A–P (*Y*)	I–S (*Z*)					
Inferior frontal gyrus	L	−40	34	26	STm	−.063	−.077	−.021	−
					DT0b	−.224	.091	.252	−
					DT2b	.783⁎⁎	.902⁎⁎	−.860⁎⁎	−
Superior parietal lobe	L	−28	−60	44	STm	–	−.126	−.273	−
					DT0b	–	.580⁎	.182	−
					DT2b	–	.371	−.650⁎	−
Angular gyrus	R	28	−60	40	STm	–	–	–	.245
					DT0b	–	−–	–	.399
					DT2b	–	–	–	.643⁎
Cerebellum (lobules I−IV)	L	−10	−42	−22	STm	–	–	–	.587⁎
					DT0b	–	–	-	.601⁎
					DT2b	–	–	–	.685⁎

⁎ *p*<.05; DT2b, dual-task 2-back; DT0b, dual-task 0-back; STm, single-task motor; IRI, inter-response interval; vel, angular velocity; asynch, absolute asynchrony; AV, average; SD, standard deviation; CRP, continuous relative phase; L–R, left–right; A–P, anterior–posterior; I–S, inferior–superior.

⁎⁎ *p*<.001.

**Fig. 6:**
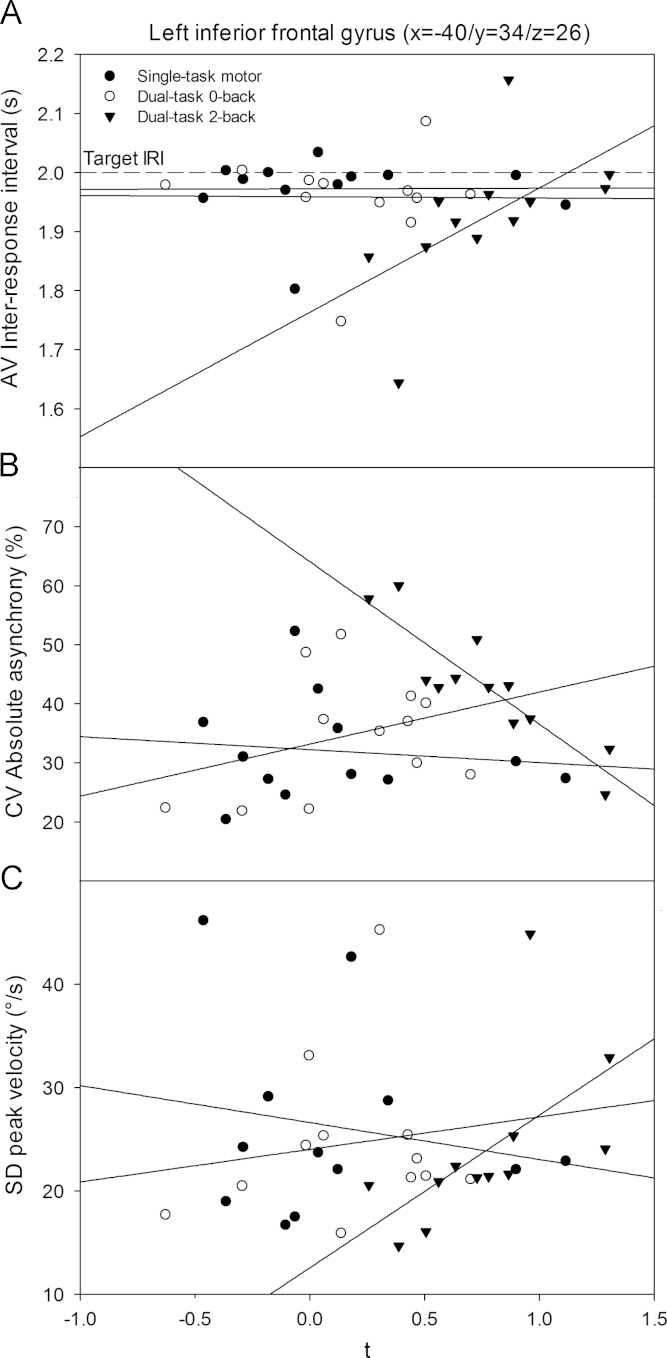
Linear regression functions between individuals' task specific local activations in the left-hemisphere inferior frontal gyrus and the (A) average IRI, (B) CV absolute asynchrony, (C) SD peak velocity for each of the three task conditions.

Fig. 6a shows linear regressions between individuals' task specific activations and average IRI for each of the three task conditions. It can be seen that in the single- and dual-task 0-back conditions, average IRI lies close to the target value of 2 s (.5 Hz) irrespective of the signal strength of the local activations. In the dual-task 2-back condition, however, low local activations were associated with faster average IRIs, whereas more accurate motor performance (closer to the target IRI value) was associated with higher local activations. Furthermore, higher local activations in left IFG were associated with lower CV absolute asynchrony, indicating less variable movement synchronization, in contrast to the other two less complex task conditions (Fig. 6b). Finally, SD peak velocity was more variable with higher local IFG activations during dual-task 2-back only, despite similar levels of SD peak velocity between the 0-back and 2-back dual-task conditions (Fig. 6c).

**Fig. 7:**
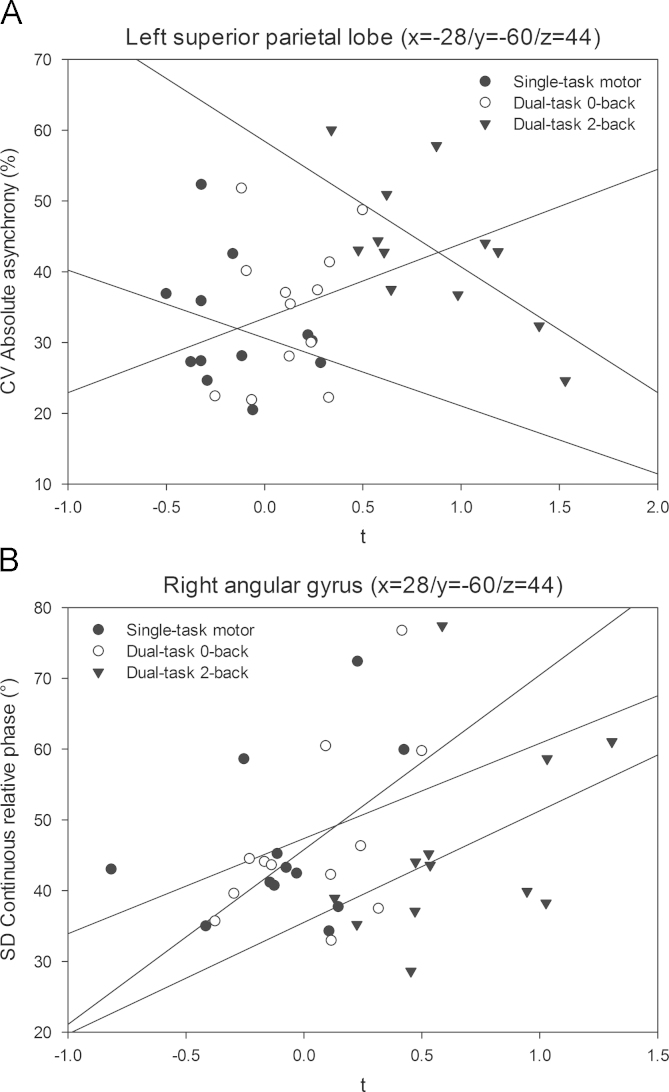
Linear regression functions between (A) CV absolute asynchrony and individuals' task specific local activations in the left-hemisphere superior parietal lobe and (B) variability of continuous relative phase and right-hemisphere angular gyrus local activations for each of the three task conditions.

Similar to the left-hemisphere inferior frontal gyrus, the ROI within the left superior parietal lobe (SPL) demonstrated a negative relationship between local activations and CV absolute asynchrony (Fig. 7a), expressing less variable movement synchronization with higher local activations. Finally, local activations within the angular gyrus of the right hemisphere were associated with SD CRP in terms of increased local activation with more variable interlimb coordination (Fig. 7b). The ROI in the left cerebellum showed similar correlations between local activations and SD CRP, however, this was common to all three movement task conditions. At the same time, the correlation between activations and in the two ROIs within the left cerebellum and the right hemisphere angular gyrus was strong (*r*=.87; *p*<.001). A similar association was not present in the single-task movement and dual-task 0-back conditions.

## 4 Discussion

We aimed to investigate the neural correlates of interference effects between the cognitive and motor domains in a cognitively demanding dual-task situation. By using a ankle dorsi-plantarflexion task, we aimed to isolate the temporal from the balance demands of gait. We expected to find increased or more widely distributed local activations representing compensatory resource adaptation in the brain to meet the timing and dual-task demands.

Our major finding is that high coordinative complexity of information processing in the dual-task 2-back condition led to an involuntary hastening of the ankle movements. Further effects of dual-task complexity were found on parameters relating to movement synchronization to the auditory pacing stimulus. Relative to the single-task motor condition, the variability of the inter-response interval increased in the dual-task 2-back condition. Similarly, the variability of absolute movement asynchrony increased relative to the other two movement conditions. The dual-task 0-back condition showed performance decrements that tended in the same direction but which statistically were not significantly different from the single-task motor condition. From this contrast between dual-tasking in the 0-back and 2-back conditions, we can conclude that externally paced, bilateral ankle movements under dual-task 2-back load involves an information processing load beyond the mere simultaneous coordination of a motor and a cognitive task. Finally, accuracy of cognitive performance was sensitive to increases in dual-task coordinative complexity and memory load. As expected, accuracy was lowest in the dual-task 2-back situation followed by the single-task 2-back and dual-task 0-back conditions.

### 4.1 ‘Hastening’ of movements under increased cognitive processing load

Our study is the first to report neural correlates of the ‘hastening’ phenomenon often observed during cognitive-motor interference. The hastening of ankle movements in the dual-task 2-back condition was negatively associated with activations found in the left IFG. Individuals who activated the IFG less strongly were more likely to deviate from the set target pace when concurrently performing the dual-task 2-back condition. At the same time, these individuals were also more likely to show more variable movement synchronization relative to the pacing stimulus (CV of absolute asynchrony), and less variable peak angular velocity. The lack of a hastening phenomenon, as well as the absence of a correlation between IFG activations and average IRI, in the dual-task 0-back condition indicates that the basic demands of coordinating two concurrent tasks are not sufficiently demanding for the effect to occur and that our participants did not display a simple direct entrainment to the pacing of the *N*-back stimuli.

The specific demands of updating working memory (not present in the 0-back condition) while simultaneously controlling the timing of ankle movements seem to be responsible for the observed interference effects. We infer that what we observed is a competition for working memory resources involved in the correction of the timing of externally cued periodic movements. At apparent odds with this conclusion is the observation that stronger IFG activations occurred with more variable peak angular velocity. However, it has been reported that movement timing is increased in accuracy by modulating a limb′s movement trajectories during the extension and flexion phases of a periodically timed movement (Balasubramaniam, Wing, & Daffertshofer, 2004). Therefore, angular acceleration and deceleration along a limb′s trajectory could result in more variable peak velocity despite less variable movement synchronization.

Involuntary hastening of the motor activity under dual-task load has also been reported elsewhere (Krampe et al., 2010, Van Impe et al., 2011). The phenomenon may be consistent with a more general pattern of regression towards a comfortable and usual movement frequency. For example, Li, Abbud, Fraser, & Demont (2012) found that older adults increased their stride-to-stride length and stride time during faster than usual treadmill walking under high cognitive load. It could mean for our study that performing the 2-back task recruited resources away from those required to maintain a slow, non-preferred frequency of movement so that some of our participants may have drifted involuntarily towards a more preferred, higher movement frequency.

### 4.2 Compensatory strategies to counter cognitive-motor interference

The observation that increased IFG activations is associated with better movement performance relates to the hypothesis of compensatory frontal activations when cognitive resources are limited. For example, Van Impe et al. (2011) examined dual-task cognitive-motor interference during unimanual continuous circle drawing and reported one ROI within the left anterior SMA that showed increased BOLD activation in the dual-task condition. They interpreted this as an adaptive upregulation to compensate for the increased demands of the dual-task situation due to structural interference in younger and older adult participants. It is worth noting that structural interference did not seem to be more severe in older adults (Van Impe et al., 2011), which validates the methodology in our present study to simulate age-related performance reductions in cognitive-motor dual-tasking.

It has been suggested that during aging, older adults shift from an automatic, subcortical mode of controlling locomotion to a mode of control demanding higher cognitive, cortical input due to a degraded mechanism of cortical inhibitory reciprocal interaction between sensory systems during locomotion (Zwergal et al., 2012). In the light of the ‘hastening’ phenomenon, we propose that delegating the control of continuous movements under dual-task load to lower-order processes might represent an adaptive mechanism of last resort for keeping performance from collapsing in a situation when higher-order resources are not available due to the additional cognitive involvement. In other words, we have described an adaptive mechanism which is involved in compensation when the demands of a cognitive task do not leave sufficient capacity to fulfil the cognitive demands of a motor task.

In contrast to the ‘posture-first’ strategy often observed in older adults in standing and walking (Maylor et al., 2001; Li et al., 2001), our study describes a compensatory strategy which gives priority to the cognitive demands in a cognitive-motor dual-tasking situation. This could mean that failure to prioritise postural control, taking into account performance decrements in the cognitive domain, during an actual locomotor or postural activity might lead to external influences becoming more likely to impact to motor behaviour, potentially leading to a fall.

In this respect, the increased fall risk in older adulthood might stem from a risky ‘re-automation’ of movement control to lower-order processes, which might be more susceptible to random external influences normally demanding higher-order compensation at the first place due to degraded intersensory ‘hedging’ as suggested by Zwergal et al. (2012). In other words, the ‘hastening’ of ankle movements could be the result of a ‘magnet effect’ (Beauchet et al., 2010) exerted by the visual stimuli, as they were presented at faster rate than the pacer stimuli. Under easy dual-task conditions this ‘magnet effect’ might be inhibited more successfully than under demanding dual-task conditions.

Although our study demonstrates increased variability of the IRI in the dual-task 2-back condition, the specific movement parameter which resembles stride-to-stride time variability most closely, it is disappointing that we were not able to identify a specific brain region of which the local activation correlated with this performance measure. It may be that the first level contrast between the dual-task 2-back condition and the fixation rest condition, which was meant to remove regions activated by visual and auditory stimuli, also removed regions that might have some involvement in the control of timed movements.

Further, it could be that hastening towards a preferred movement pace and the magnet effect are not mutually exclusive. A reduction of available cognitive control resources could result in drift as well as vulnerability to involuntary synchronization with the secondary task. Future studies would need to explore different frequencies in the secondary cognitive task relative to the frequency of the primary movement pacing while holding the cognitive demands constant to deconfound these alternative interpretations.

### 4.3 Production of externally paced periodic movements

The results of our study are in good correspondence with observations that active periodic ankle movements reliably result in BOLD activations in the primary sensorimotor cortex of the paracentral lobule, the premotor cortex, the supplementary motor area, the cerebellum, the thalamus, the secondary somatosensory cortex, as well as the superior temporal gyrus (Ciccarelli et al., 2005, Dobkin et al., 2004, Francis et al., 2009, MacIntosh et al., 2004, Newton et al., 2008, Trinastic et al., 2010, Rocca and Filippi, 2010). In addition, these ankle-specific activations are also in good correspondance to a recently published study by Toyomura, Shibata, and Kuriki (2012), who investigated the neural correlates of the production of bilateral leg movements around the knee joint and found activations within the same regions. This gives us reason to believe that our bilateral ankle movement task tapped into the supraspinal locomotor control network.

With respect to the requirements of synchronizing discrete movement with an external auditory pacing stimulus, Rao et al. (1997) found that synchronization of finger tapping activated the left-hemisphere sensorimotor cortex and parts of the cerebellar right hemisphere, while Lewis, Wing, Pope, Praamstra, & Miall (2004) argued that monitoring and adjusting tapping movements in response to perceived asynchronies between taps and pacer involves frontal and prefrontal regions as well. It may be, however, that at least in the dual-task 2-back condition regions within the left superior parietal lobule are also involved in timed synchronization in addition to the inferior frontal gyrus. Thus, our findings indicate a functional relationship between the IFG and the SPL in the left hemisphere in the context of synchronizing bilateral ankle movement timing to an external auditory pacing signal.

### 4.4 Bilateral interlimb coordination

The variability of interlimb coordination in our present study was not affected by the specific dual-task demands. This is perhaps surprising as it has been demonstrated that the variability of a bimanual coordination pattern under dual-task load follows an U-shaped function over the range of movement frequencies with increased dual-task costs at both ends (Zanone, Monno, Temprado, & Laurent, 2001). In addition, slow walking has been reported to result in more inconsistent anti-phase interlimb coordination of the legs compared to normal or fast walking, which might suggest greater cortical involvement and emphasised supraspinal input in slow walking (Plotnik, Bartsch, Zeev, Giladi, & Hausdorff, in press). Our current observations might represent a ceiling effect due to the slow pace of ankle dorsi-plantarflexion performed in our study.

Despite these behavioural findings, we uncovered an association between SD CRP and activation within the right hemisphere angular gyrus in the demanding dual-task 2-back condition. It appears remarkable, however, that the variability of SD CRP increased with greater activation of the right angular gyrus, suggesting that activity in the right angular gyrus did not improve the stability of interlimb coordination. On the other hand, a relationship existed between the right angular gyrus and a region within the left cerebellar hemisphere (lobuli I–IV), which showed high correlations between local activations and SD CRP in all task conditions involving movement production. It may be reasonable to conclude that the right angular gyrus was activated in concert with the left anterior cerebellar hemisphere as an auxiliary circuit for the coordination of slow bilateral continuous anti-phase ankle dorsi-plantarflexion movements during the dual-task 2-back load. Hypothetically, without involvement of the right angular gyrus, SD CRP might have increased in the dual-task 2-back condition beyond the level of the single-task motor and dual-task 0-back condition.

It seems remarkable that the findings in our study emphasise the involvement of left hemisphere regions within the IFG and SPL. This finding stands in contrast to reported activation increases in a network of areas within the superior parietal and dorsal premotor cortices primarily in the right hemisphere during anti-phase bimanual movements (Wenderoth, Debaere, Sunaert, van Hecke, & Swinnen, 2004) as well as wide-spread activations of bilateral cortical areas within the frontal, temporal, and parietal lobes during bimanual tapping (Ullén et al., 2003, Wenderoth et al., 2005). Although periodic bimanual coordination seems to result in activations of the same primary and secondary motor areas as unimanual movements (Walsh et al., 2008), quantitative and qualitative differences between uni- and bimanual movements were also reported. For example, bimanual movements seem to alter the modes of interhemispheric interaction between homologous motor areas and the motor dominant hemisphere as the primary driver (Walsh et al., 2008). As all our participants were left hemisphere dominant, this might explain the finding of left lateralized ROIs related to dual-task ankle dorsi-plantarflexion movements.

### 4.5 Lateralization of movement parameter-related brain activations

A subvocal rehearsal strategy suggested by the secondary cognitive task could also explain the strong left lateralization found in our results which contrasts with more bilateral or right lateralized mechanisms for timing and interlimb coordination. For example, Van Impe et al. (2011) combined periodic mental arithmetic with a unilateral movement task that imposed explicit spatial constraints (bounded circle drawing) and Wenderoth et al. (2005) investigated bimanual activity imposing spatial demands on participants’ movements.

Intrinsic facilitation of inhibitory control processes by subvocal rehearsal in the 2-back condition might be one reason why hastening of movements was prevented in participants with greater IFG activation. Badre and Wagner (2006) reported that left hemisphere mid-ventrolateral prefrontal cortex (mid-VLPFC; inferior frontal gyrus pars triangularis) activation increased with increasing proactive interference in a task switching paradigm. Thus, either more intense subvocal rehearsal or more effective conflict resolution in the 2-back task is associated with greater local IFG activations, which then facilitates the control of movement periodicity in terms of structural interference within the prefrontal cortex. Our current data, however, are not entirely conclusive with respect to this argument, as each of the three movement parameters did not correlate with accuracy in the cognitive task.

### 4.6 Dual-task specific correlates for the coordination of a movement task and a cognitive task

As stated earlier, we did not make firm predictions as to whether we should observe ROIs specific to dual-task conditions beyond those implicated in the single tasks alone. The interaction contrasts between the dual-task 2-back condition and the sum of the two relevant single-task conditions in our study did not reveal any emergent dual-task specific local activations. A similar null finding was also true for the dual-task 0-back interaction contrast. In this respect, our study is in line with previous literature indicating the lack of dual-task specific local activations for cognitive-motor dual-task paradigms (Johansen-Berg and Matthews, 2002, Van Impe et al., 2011). This contrasts with cognitive-only verbal-spatial dual-task paradigms, however, for which dual-task specific activations have been reported (D’Esposito et al., 1995). The first reason for not finding dual-task-specific activations may be lack of statistical power. Second, it may be that although we aimed to create a situation with heavy dual-task processing load, participants were able to carry out both concurrent tasks well enough so that the task-relevant brain regions were sufficient to meet the dual-task requirements. Third, it may be reasonable to conclude that the degree of structural interference in cognitive-motor dual-tasking is less than in cognitive only dual-tasking.

## 5 Conclusion

Our study investigated the neural correlates of the temporal control aspects of gait, such as stride-to-stride time, in isolation from the biomechanical and postural demands of walking. We observed reduced activations within regions of the left hemisphere inferior frontal gyrus, and in some respect also within the superior parietal lobule, as a locus of involuntary ‘hastening’ and more variable external synchronization of ankle movements while being distracted by heavy dual-task working memory load. To the extent that our dual-task results generalise to healthy older adults, it may be that involuntary changes in cadence in older adults are caused by the failure to sufficiently activate the left inferior frontal gyrus. Those individuals who do activate left IFG to a greater amount may be able to optimise their gait timing in the face of reduced cognitive and physical abilities.

Future functional neuroimaging studies of older adults during timed movement in isolation and during actual walking are needed to more directly address this issue. The results of our study also suggest the left IFG as a target region of non-invasive brain stimulation in combination with cognitive training (Bentwich et al., 2011; Schulz, Gerloff, & Hummel, 2013) for the purpose of ameliorating impaired cognitive-motor dual-tasking in older adults or specific patient populations with neurological diseases such as Parkinson's and Alzheimer's.

## Conflicts of interest

None.
